# “Scalping” in the context of criminal dismemberment and mutilation—a case report and review of the literature

**DOI:** 10.1007/s12024-023-00581-x

**Published:** 2023-02-03

**Authors:** Stefan Potente, Sara Heinbuch, Frank Ramsthaler, Nadine Schäfer, Nadja Walle, Peter Schmidt

**Affiliations:** 1 Department of Legal Medicine, Medical School, University of Saarland, Kirrberger Strasse, 66421 Homburg/Saar, Germany; 2 Department of Toxicology, Medical School, University of Saarland, Kirrberger Strasse, 66421 Homburg/Saar, Germany

**Keywords:** Scalping, Scalp-taking, Dismemberment, Mutilation, Mohawk

## Abstract

We report on a case of criminal dismemberment and attempted scalping of a homicide victim with a “Mohawk” haircut. Case findings are presented. A review of the literature was performed for scalping in its historical and cultural context and particularly in criminal dismemberment and mutilation: Historically, scalping was prevalent in many ancient cultures around the world, where scalps were taken as trophies or “proof of kill”, much like shrunken heads, trophy skulls, and other artefacts. Scalping was particularly widespread in Northern America in the context of tribal warfare, both before and after colonization. The iconic “Mohawk” haircut is closely linked with scalping, as it was meant to taunt the enemy. In the modern forensic context, scalping constitutes a form of criminal mutilation. However, cases of criminal dismemberment and mutilation are rare in forensic casework. Our literature review revealed a low number of scalping in criminal dismemberment and mutilation cases. The documentation was overall poor. Positioning scalping within the classification of criminal mutilation and dismemberment was difficult. In literature, even though case numbers were small, the majority of “textbook scalping” cases were German. The presented case, to our best knowledge, is the first modern-day photo-documented case of (attempted) scalping, even more so of a person wearing a “Mohawk”.

## Introduction

We report on a case where a “Mohawk” wearing modern person was subjected to attempted scalping in the context of criminal dismemberment and mutilation. In addition, a literature search on the phenomenon of scalping in the forensic context was performed.

“Scalping”, in the general medical context, describes the (partial) detachment of a person’s scalp from the skull.^1^ Accidental scalping may occur when hair gets entangled in rotating parts of machinery or when the scalp is sheared off by direct, shallow-angled blunt force [1]. Also mauling by dogs and other animals may involve scalping [2]. Clinically as well as in extensive trauma, the terms “defleshing” (for example in [3]) or “scalp avulsions” are preferred (for example in [4]). The detachment of the scalp from the skull without external wounds in the context of child abuse by hair pulling has been dubbed “scalping” in German-speaking articles in the adoption of radiological terminology [5, 6]. However, the term subgaleal haematoma may help to avoid the confusion of this phenomenon with open wound scalping.

Scalping/scalp-taking as an ancient practice was prevalent—as described in more detail in the respective sources—in ancient China, South America, Central Europe, Scandinavia [7], and Egypt [8]; it was performed by the Scythians (as described by Heredotus [9], according to [10]), possibly Roman legionaries [11], and others.

Despite this, it “is generally, but falsely, supposed that only Americans scalp” [12]. Scalping/scalp-taking is often viewed as “the iconic mutilation practice of the nineteenth-century American West” [13].

North American scalping was in fact commonplace, with the exception of the Inuit [14]. North American scalp-taking was prevalent in armed tribal conflicts well before European contact, supported by “sufficient archeological evidence” [15]. Some contributing factors to the widespread practice of scalp-taking in North America before the arrival of Europeans include the following:

- “Raid” warfare with small groups of warriors attacking quickly, without accountable witnessing or body counts.
- Warriors’ role in tribal society, needing to prove themselves in battle; with the easy fabrication of trophies from scalps by drying and stretching them on a wooden hoop.
- Practical transportation during raids and nomadic life favours scalping over taking whole heads.
- Spiritual transfer of powers from the victim to the scalp-taker (scalp considered to contain the soul) [7, 13, 16] and denial of entry to the afterlife for the scalped enemy through bodily incompleteness [17].

One example is the Crow Creek massacre of around 1300 AD [17–19]. A discovered mass grave, later described as a “Pol Potian kind of landmark” [16], contained the skeletons of 500 men, women, and children. Almost all had been mutilated, tortured, and scalped. After the arrival of Europeans, especially in the course of the armed conflicts which followed and which involved varying factions of Europeans and native tribes fighting each other, scalping was systematically promoted as “proof of kill” by paying a considerable bounty [13].

**Fig. 1:**
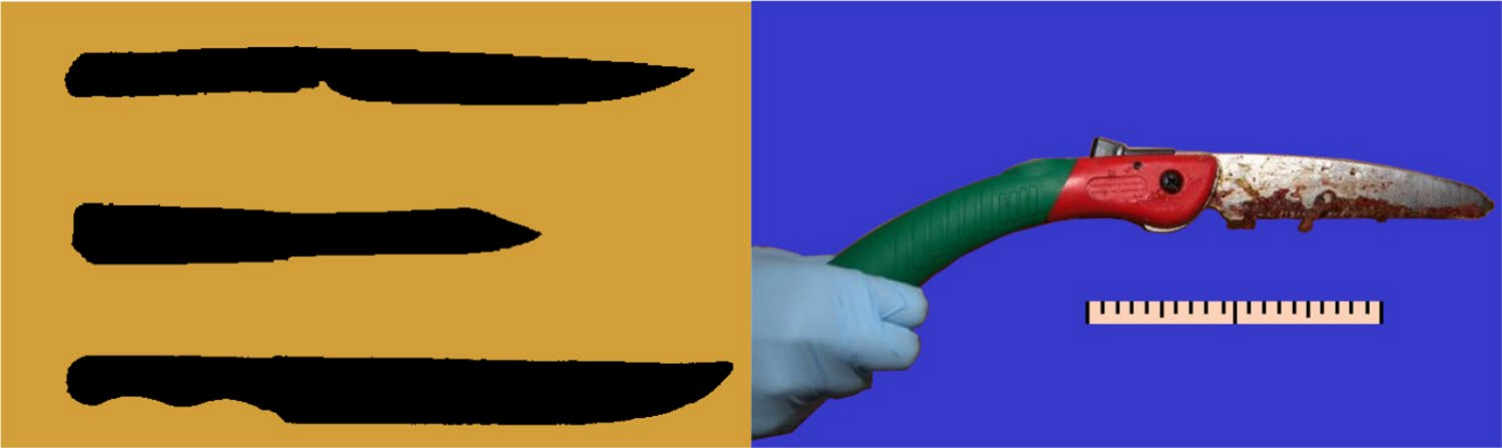
Left: Silhouettes of alleged “scalping knives”. Top: National Museum of Scotland [26]. Center: Karl-May-Museum in Radebeul, Germany ([27]). Bottom: John “Liver-Eating” Johnston’s knife [28]. Right: foldable hand saw, likely used in scalping attempt in our case

It is unclear when (and if at all) scalping has finally seized in armed conflicts. According to unconfirmed claims, US and Canadian soldiers of native American descent have performed scalpings in both World Wars [16], and scalpings have been performed in conflicts as recent as the Korean and the Vietnam war. Tools for scalp-taking were initially primitive, such as sharp obsidian rocks, before metal knives became widespread on the American continent. These general-purpose knives were used for scalping. There were probably no dedicated “scalping knives”, even though many knives have been claimed to be such. However, some knife features are particularly well suited for running a blade around the skull. These include a medium length to long, straight to a slightly rounded blade with a sharp tip, often without a hand guard. Such knives include Bowie knives, used for example by John “Liver-Eating” Johnston^2^ or Buffalo Bill^3^ (see Fig. 1).

Regarding the technique of scalping in North America, Burton writes the following: “Scalp-taking in America is a solemn rite. In the good old times men scrupulously awaited the wounded man’s death before they ’raised his hair’ [...] When the Indian sees his enemy fall, he draws his scalp-knife [...] and twisting the scalp-lock, which is left long for that purpose and boastfully braided or decorated [...], round his left hand marks with the right two semi-circular incisions, with and against the sun, about the part to be removed. The skin is next loosened with the knife point [...] The operator then sits on the ground, places his feet by way of leverate against the subject’s shoulders, and holding the scalp-lock with both hands, he applies a strain which soon brings off the spoils, with a sound which, I am told, is not unlike ’flop’. Without the long lock it would be difficult to remove the scalp.” The author notes, that different tribes had different fashions of scalping, and the Sioux tribe, for example, removed the whole head skin, including a portion of the ears [12].

Observable anthropological evidence of scalping on skulls consists of “cuts typically most prominent across the fontal, near the level of the hairline and more sparsely across the parietal, while often absent across the occipital, presumably because the scalp had been pulled loose from the bone at that point to be cut free” [15]. More generally, and seemingly ubiquitous to cultures with a tradition of scalping, are superficial cuts on the cranium in circular, lengthways and parallel orientation, seen, for example, in ancient Egypt [20] and in Fifth century Italy (likely committed by Huns) [21].

Survival was rare, since scalping was performed mostly on the dead and dying. Scalping is in itself not immediately lethal, but associated with a very high rate of complication. Some skulls from the Crow Creek mass graves (see above) show signs of healing/vital reaction [17]. Medical treatment, at least for some eighteenth-century scalping survivors on the American frontier, was available in the form of a surgical technique developed by Belloste in 1696. It has been re-introduced as a neurosurgical procedure only relatively recently [22]. The “Mohawk” hairstyle, named after the “Mohawk” tribe as the most easterly section of the Haudenosaunee (Iroquioian Confederacy), is closely linked with scalping in North America. Cutting, shaving or plucking the hair short on both temples leaves a long pluck of hair in the center. Such hairstyle was most often worn by warriors in times of conflict. Here, it was used to taunt the enemy and display readiness to the consequences of the fight. Different skull defects between adults/adolescents and children of the “Crow Creek Massacre” have been speculated to be caused by children’s style of “Mohawk” hair, which may have caused smaller proportions of the scalp being taken [23].

The “Mohawk” hairstyle has found its way into western (pop) culture, arguably when American paratroopers in World War II shaved their heads to appear more frightening, since “much of the German army believed that American airborne divisions comprised of criminals, murderers, and psychopaths” [24]. It was later incorporated into the “punk” subculture. Many variations exist, such as the “faux-hawk” (simulating a Mohawk using hair gel on the temples) and “dread-hawk” (combination with short dreadlocks). The hairstyle is however not unique to North America. Similar hairstyles have been identified in a male “bog body” from Iron Age Ireland (the “Clonycavan man” [25]) and the traditional Ukrainian “Cossack” hairstyle. Also, different religious, particularly Hindu, hairstyles may constitute shaven heads with central isolated locks.

## Case

A member of the “punk” subculture was drinking alcohol with two acquaintances in an apartment. A dispute broke out and the two started beating and kicking the man. They mocked him by smearing spray paint on his face and dressing him up. Then, they killed him with a combination of sitting on the chest, kneeling on the neck and pressing a pillow on the face.

The perpetrators proceeded with dismembering the body, using various tools such as hacksaws, a power saw, a box cutter and a foldable hand saw (see Fig. 1, right). This procedure took several days. Removed limbs and body fragments were packaged into plastic trash bags. Some of them were taken to the woods by means of a small two-wheeled shopping trolley and buried, while others remained in the apartment along with the torso. The perpetrators finally attempted to further dismember the torso. When one of the offenders attacked and injured the sleeping other, the latter informed the police.

The body was found roughly 1 week after the killing. The exact time of death could not be precisely established. One defendant claimed she had intended to “slice the head up” but failed. Both the accused were sentenced for manslaughter (§ 212 German Criminal Code).

**Fig. 2:**
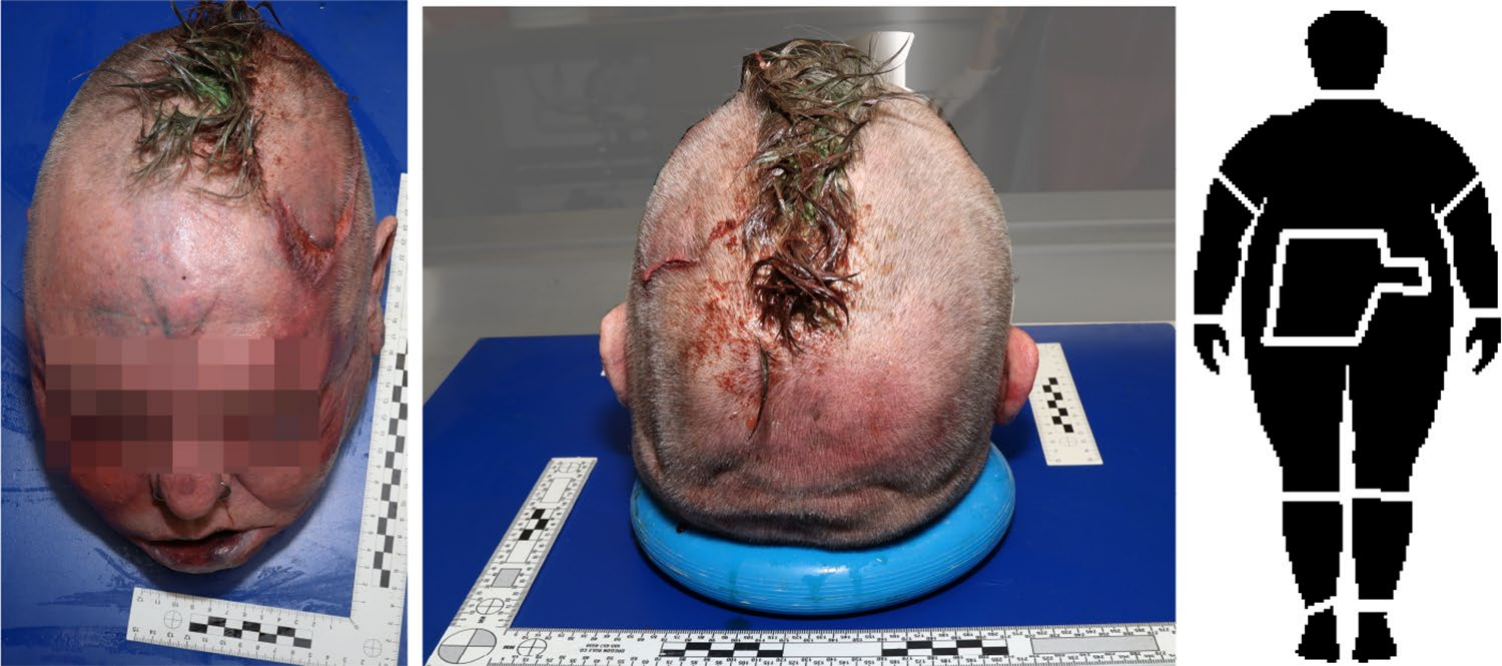
Severed head (after cleaning). Left: frontal view. Note fine cuts and remnants of spray paint on the forehead. Center: posterior view. Right: pattern of dismemberment. Note excision of the abdominal wall

**Fig. 3:**
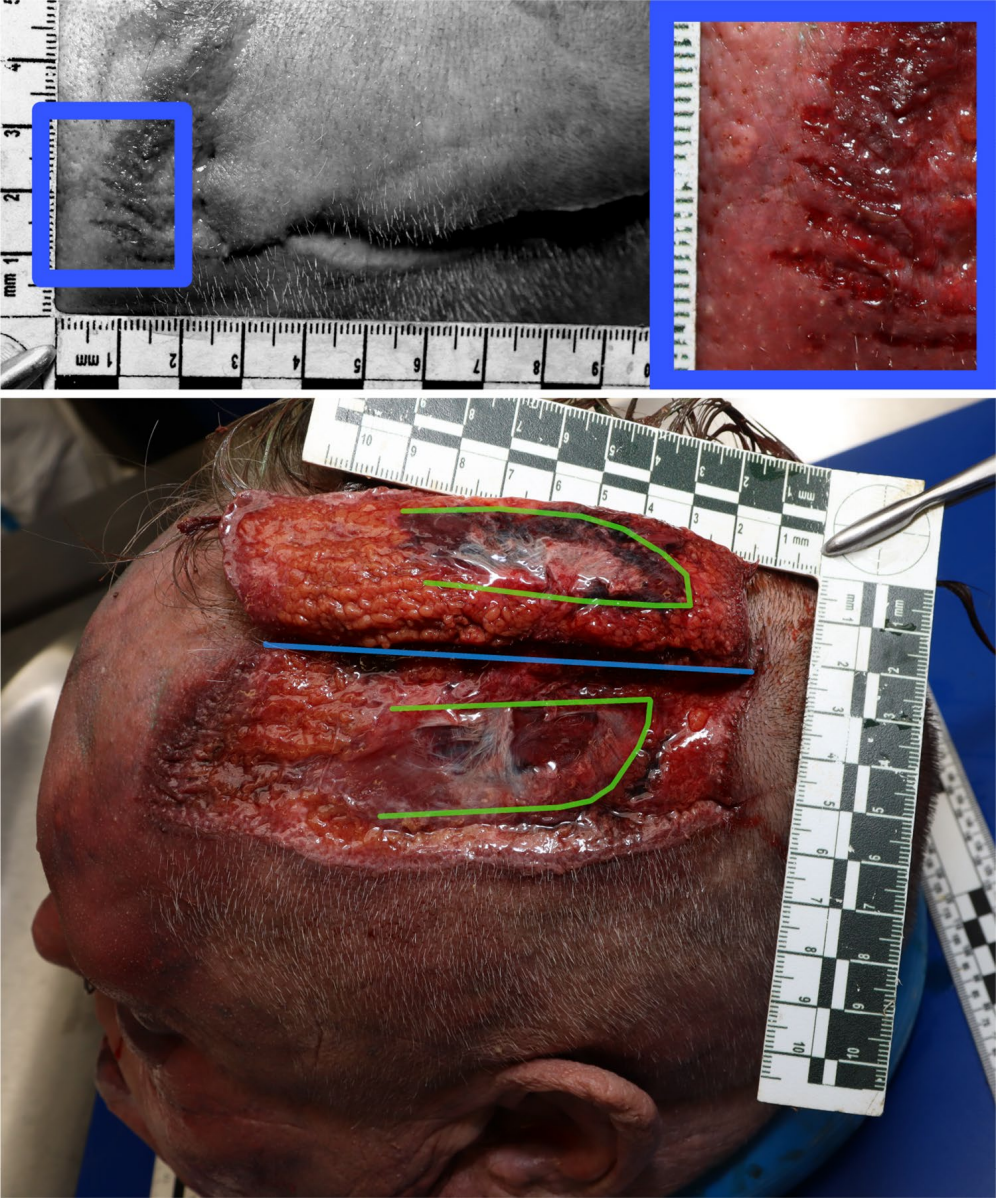
Top: anterior wound edge (overview and detail). Bottom: scalp flap showing haemorrhage and visible blade shape in wound cavity

**Fig. 4:**
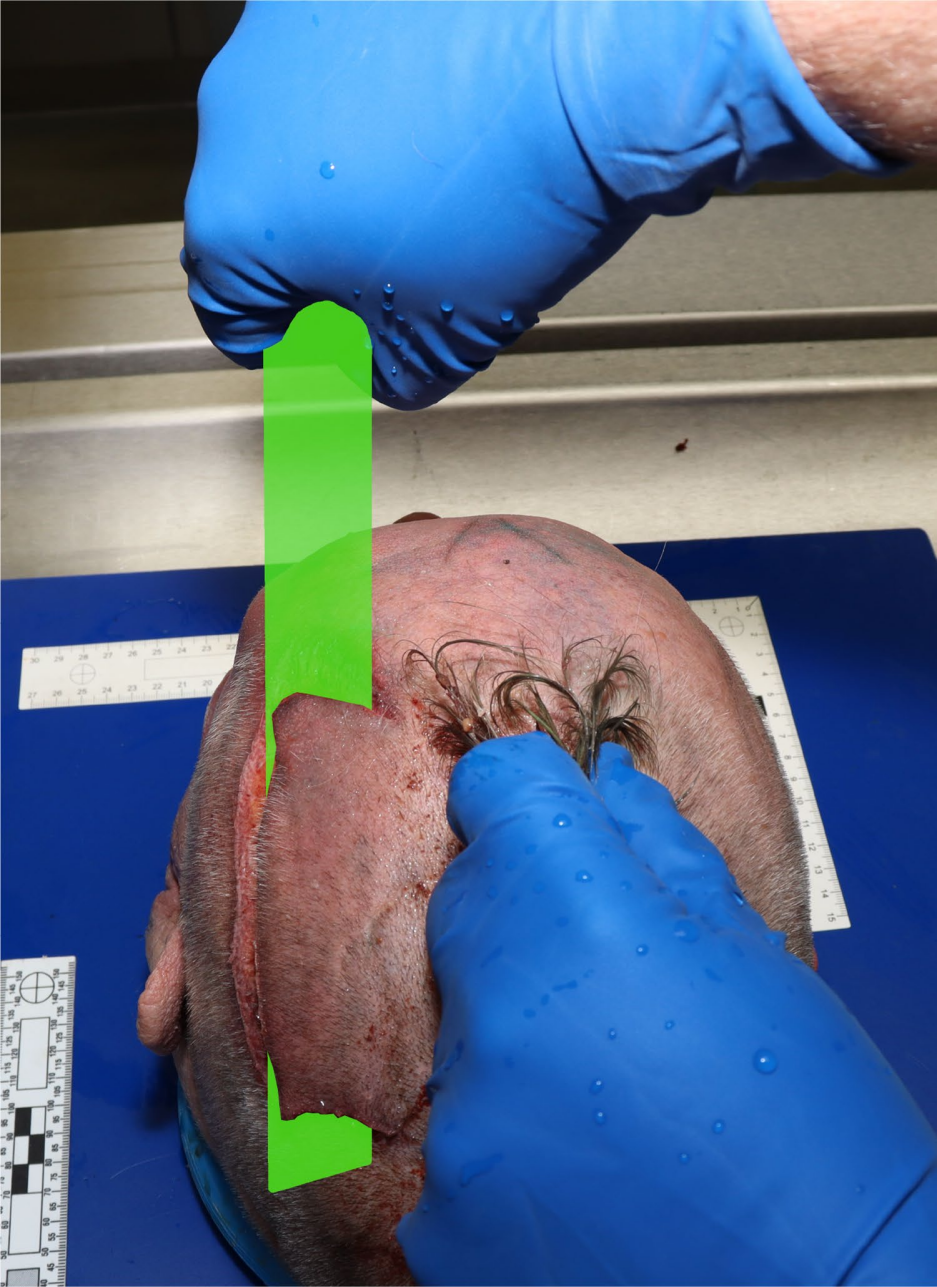
The demonstration of possible scalping procedure during autopsy with digital superimposition of a “blade”. Note the gripping of hair

Autopsy findings (also see Figs. 2, 3 and 4) are as follows:

- 60-year-old, morbidly obese male: body height approx. 164 cm, body weight 119 kg (BMI 44.2). Multiple tattoos, remnants of greenish paint on forehead.
- “Mohawk” haircut, with hair on the temples shaved off and the remaining strip of hair dyed bright green. Partial scalping of the left temple in horizontal orientation, with deep tissue incision. A “blade” shape was recognizable in the wound cavity which also displayed haemorrhage. On the anterior wound edge, “ladder” shaped defects, oriented along the wound axis were encountered.
- Fine superficial cuts on the forehead and temple.
- Petechial haemorrhages of the conjunctivae and inner surfaces of lips and gums. Soft tissue haemorrhage of the face and left temple. Haemorrhage and lacerations of lips. “Grip marks” on the inner aspects of both upper arms. Soft tissue haemorrhage in projection on the upper spine and scapulae.
- Dismemberment with detachment of body parts into a total of 10 fragments, including the torso, head, arms, hands, lower thighs, feet. Removal of a Q-shaped piece of abdominal wall with access to the abdominal cavity. Removal of a 678 g piece of liver (total liver weight 3602 g), and pieces of the small intestine. No additional circumscribed mutilation were present (such as detachment of nose, ears or genitals).

Toxicological analysis was performed in parts to identify the concentrations of prescription drugs in light of death time estimation (last time of intake known). Besides the prescription drugs amlodipine, olanzapine, pipamperone, quetiapine, sitagliptin, acetylsalicylic acid and ibuprofen, chemical-toxicological analyses revealed the presence of amphetamine (traces), diazepam (sub- therapeutic range) and chlorprothixene (sub-therapeutic range) in femoral blood. Results were inconclusive in regard to death time estimation.

## Literature search

A literature search for “scalping”, “scalp-taking”, “skinning” and “defleshing” in the forensic context, particularly in criminal dismemberment and mutilation (cDM), was performed on the websites PubMed and Researchgate, as well as in the authors’ personal collection of articles and available textbooks. Raijs et al. define dismemberment as “amputation of a limb or portion of it” and mutilation as “the act of depriving an individual of a limb, member, or other important part of the body; or deprival of an organ; or severe disfigurement” [30]. “Scalping”, as severe disfigurement, qualifies as mutilation.

In the electronic format, a full-text search was performed on all articles to retrieve cases not included in keywords, title and abstract. Accidental or otherwise general medical “scalping” had to be filtered out, as were articles focused solely on bone damage, such as [29]. Studies which provided insufficiently detailed description of cDM patterns were nevertheless used for data on the prevalence of cDM. Using these filters, only a few publications remained (see Table 1).

**Table 1:** Survey of publications. Case count (*N*) includes all cDM cases in the respective publication, regardless of the possible absence of the head for inspection

*N*	Authors/publications	Notes
31	Püschel and Koops [31]	Hamburg, Germany. Note case
18	Adjei [32]	Overlap
51	Wilke-Schalhorst et al. [33]	
2*	Edler et al. [34]	*Cases from [34] included in [33]
30	Konopka et al. [35]	**Case from [36] included in [35]
1**	Kunz and Gross [36]	
22	Rajs et al. [30]	
43	Guggenheimer et al. [37]	
65	Sea and Beauregard [38]	
13	Häkkännen-Nyholm et al. [39]	
40	Mata-Tutor et al. [40]	
3	Di Nunno et al. [41]	
1	Weimann [42], Stekel [43]	Sources [42, 43] almost identical
	Berg [44]	
	Black et al. [18]	Books
	Wirth and Schmeling [45]	

Positive cases for “scalping” were overall sparse:

- A 2010 dissertation [32] from Hamburg, Germany, reports on 18 cDM cases covering the period from 1985 to 2008. In six out of 18 cases, the head was not available for inspection. “Scalping” is noted for “Case 13”. The body was found cut up into 11 larger pieces in two barrels on the perpetrator’s balcony in advanced putrefaction, approximately 4 months after death. The trunk was severed into two halves. Arms, hands, legs and head were severed, too. The scalp of the parietal and frontal region, with auricles attached, is listed as one fragment. The mandible with tongue and soft tissue attached is listed as another. Dismemberment was started 1 week after death with the noticeable onset of decomposition present.
- Out of 30 cDM cases from Krakow, Poland, between 1965 and 2015 [35], the head was still attached in only two cases. No precise information is given about how many heads were available for inspection. Two cases are described to have been burnt, a third partly charred, with the soft tissues divided into 770 parts. In another case, only the torso was examined.
  Overall, two cases of scalping/skinning were identified in the article: In the first case, the offender killed his wife, severed and shaved her head and skinned the entire face. In the second, more bizarre case (also published separately in more detail in [36]), the scalp was removed by a circular cut which was then extended across the face. The offender, the victim’s son, then proceeded to sew the tissue into a mask which he wore in front of his grandfather.
- Rajs et al. presented 22 cDM cases from Sweden (1961 to 1990) [30] with 20 discernible cDM patterns. In 17 cases, the body was decapitated, and in five cases, the head remained missing. Two cases included scalping. In “case III:4”, the female victim was killed by drowning in the bathtub, then scalped and skinned. Both eyes and jaws (including teeth) were removed as well as internal organs and muscles. The male perpetrator, who prepared and ate at least 10 meals out of the dismembered body, suffered from schizophrenia. In “case III:6”, a young man was killed by manual strangulation in combination with the cutting of the throat while incapacitated. The perpetrator, who also suffered from schizophrenia, then scalped the head and removed both eyeballs, teeth and jaws as well as the genitals.
- The prominent 1935 Ruxton case (also known as the “Jigsaw Murders”), as described in [46], which famously premiered the technique of photographic superimposition for forensic identification, included two victims which were found dismembered and decomposed in a ravine. The victims’ eyes, ears, nose, lips and facial skin had been removed by the perpetrator, who was a medical doctor, primarily to hinder identification. One body (the perpetrator’s wife) was in addition to the above-mentioned injuries scalped, and terminal segments of the fingers were removed. The other body (housekeeper) was not scalped, but a large piece of scalp was missing.
- In the book “Criminal dismemberment: forensic and investigative analysis” by Black, Rutty, Hainsworth and Thompson [18], mainly pre-historic and “tribal” aspects of scalping are covered. One historic case, positive for “scalping”, is included:
  The yet unsolved “Thames Torso Murders”, from late nineteenth-century London, include one case, in which a female victim’s face with was found with the scalp attached. The chin and a portion of the mouth had been cut from the face; the nose was sliced but still attached to the upper lip - by description reminiscent of facial trauma depicted in Püschel and Koops [31]. Interestingly, a hoax confession letter later claimed its author had “scalped her in the Indian fashion”.
- The book “Kriminelle Leichenzerstückelung” (“criminal dismemberment”) by Wirth and Schmeling [45] lists 448 isolated cases of cDM from mainly German-speaking journals, books and dissertations spanning more than a century. Out of those, “scalping” was described in only a fraction of cases: German serial killer Willy Wenzel killed a total of five people in Jena, Germany, in the 1920s. Two cases involved cDM, including cutting out the genitals of a 10-year-old girl after stabbing her. The other case, his former girlfriend, was found buried, dismembered and scalped, with her braid attached to an “American scalping-knife”. Even though Wenzel committed suicide soon after his arrest at the age of 27, some details of his life leading up to the crimes were uncovered. He had been living a fantasy as a leader of a group of younger adolescents who dressed up and role-played as “American Indians”. He collected tribal paraphernalia and staged photos which depicted him as a cruel savage performing acts of torture. His followers addressed him as “Chief Bosko” and Wenzel signed interrogation reports as such. At work, he was mocked as he was “jumping around the factory locker room with a scalping knife clenched between his teeth” [42, 43]. Another case included the killing of a juvenile co-worker after sexual assault by throttling with a cable, followed by cDM including the removal of organs from the chest and abdomen, removal of genitals and scalping of the head without apparent haemorrhage, cited as “Case 22” from [47] (not available for the current publication). In addition, the book presents two cases from Rajs [30] (see above) and Berg’s “Case 60” [44] (see below).
- In his book “Das Sexualverbrechen” (“Sexual Crime”), Berg describes 64 cases of different sexually motivated crimes from the viewpoint of forensic pathology (including, but not limited to, homicide) [44]. “Case 60” describes the killing of a 23-year-old woman in Germany in 1950. The “hairy skin of the head was removed in the fashion of scalping, so that the bloody skull was exposed”. The body was tied around the knees and the neck. The photographic documentation of the removed scalp confirms the circumferential cut around the head with a “typical” specimen of the removed scalp. The perpetrator claimed he had intended to fabricate a wig from the removed scalp. “Case 61” describes multiple sexually motivated homicides by the Munich serial killer and rapist Johann Eichhorn, who between 1928 and 1939 committed at least 100 rapes and five sexually motivated homicides on women. Three of his homicides involved cDM in the form of cutting off the legs and cutting out the genitals. Eichhorn quoted the following: *“I went all crazy when I touched the hair [.] I cut away the hair”*. However, concerning the context, it is likely that he was talking about pubic hair, not scalping.

Negative or undetermined for “scalping” cases are predominant in cDM cases published in literature:

- Amongst 31 cases of cDM from Hamburg, Germany, 1959 to 1987, facial mutilation was present in several cases, mainly directed at the ears, nose or lower half of the face, but no distinctive scalping pattern. Scalping was notably absent for eight cases of “sexual perversion” as well as two cases of “necrophilia” [31]. Note the partial overlap of observation times/cases with [32–34].
- Out of 51 cDM cases from Hamburg, Germany, 1959 to 2016, 21 were classified as “offensive”. However, no particular cDM patterns were presented [33]. It was mentioned that in 10 cases, the pattern was incomplete due to missing body parts. Two cases out of these 51 were later published separately in more detail and did not include scalping [34].
- In a study on 40 cDM cases from Spain, scalping was not recorded as a variable. However, two cases of face mutilation were present [40].
- In a study on 65 cDM cases from Korea, scalping was not specified as a variable. Modus operandi was however closely surveyed, including sexual intercourse with the corpse (*n* = 1) and dousing the body with acid or another chemical (*n* = 3) [38].
- Another study from Sweden, following up on cases between 1991 and 2017, presented 43 cDM cases. No detailed cDM patterns were given. It is mentioned the cause of death could not be established for all “defensive dismemberment” cases because of the absence of body parts, decomposition or destruction. No case of scalping was identified. However, authors noted a rise in cDM percentage amongst homicides in Sweden from 0.5% in the 1960s to 2.4% in the 2010s [37].
- Out of 13 cDM cases from Finland in an offender-centric study, representing 2.2% of homicides between 1995 and 2004, two cases of “offensive” mutilation were identified without further specification of patterns [39].
- Amongst three well-documented cDM cases from Italy (with one head missing), no scalping was present [41].

## Discussion

Scalping is difficult to fit into existing cDM classification. Traditionally, the classification of cDM, for example in [31], distinguishes between either “offensive” or “defensive” dismemberment. In their 1998 article, Rajs et al. used an expansion of this classification [30]:

- Type I (“defensive mutilation”), where the offender aims at successful disposal of the body and/or hindrance of positive victim identification.
- Type II (“aggressive mutilation”), where the act of killing triggers excessive violence which includes mutilation of the body and which may include the face and/or genitals.
- Type III (“offensive mutilation”):
  - IIIa: necrophilic desire to kill and perform sexual acts on the body.
  - IIIb: sexual-sadistic urge to perform sexual acts and inflict pain and injury during the killing, with mutilations being initiated when the victim is still alive (alternatively, such actions can only begin after the victim’s death).
- Type IV (“necromantic mutilation”): actions are performed on the dead victim only, with either true necrophilia or the wish to retain trophies, symbols or fetishes.

“Simulation” (to create a “false lead”) has been proposed as yet another class of dismemberment. In one example case cited in [45], the offenders tried to simulate a sexually motivated crime by cutting off the penis of the victim after killing him.

By the given definitions, scalping may fit into almost all classes, depending on the case characteristics:

- Type I, when combined with (or extending to) skinning of the face to hinder identification.
- Type II, when excessive violence is aimed at the head and face.
- Type IIIb, when scalping is started as a non-lethal method of torture on the living victim.
- Type IV, when the scalp is taken as a trophy.

One might, when applicable, even discuss Type IIIa in the context of piquerism, “the sexual inclination to stab, pierce, or cut – obtaining sexual gratification from the shedding of blood, tearing of flesh, and / or observing such pain and suffering of a victim who is subjected to this activity” (as defined in [48]).

As pointed out by Wirth and Schmeling [49], there are intermediary cases which fall between “offensive” and “defensive” dismemberment. The destruction of the body may not only be seen as a continuation of aggression against the victim, but may also aim at disfigurement “beyond recognition”, which is indicative of a “defensive” motivation for dismemberment. Furthermore, given the rarity of events and the individual circumstances, the isolated view on either findings or case history may lead to oversimplified conclusions regarding classification.

Consider for example a case published by Karger, Rand and Brinkmann with a distinctive cDM pattern in a young woman: the hands had been cut off, and the female genital tract had been “cut out”. The cDM pattern suggests offensive, aggressive or even necromantic cDM. It was however in fact defensive: the perpetrator had tried to hinder successful DNA investigations after having sexual intercourse with the dead body of the woman he had just strangled [50]. Regarding the overall occurrence of scalping and “missing” data, scalping for the sake of scalp-taking, as an act of deliberate mutilation, qualifies as cDM.

Cases of cDM are in themselves rare and constitute low single-digit percentages of total homicides. Since cDM is performed defensively in a majority of cases to prevent the finding of the body, a significant number of unreported cases are to be expected.^4^ Relevant for scalping, hiding of the body may only partially succeed, and a non-negligible number of heads were missing in literature case collections (see above). Without a head, scalping can of course not be diagnosed. Accordingly, excessive violence involving the face as a feature in “aggressive mutilation” (Type II) cannot be evaluated either, when the head is missing. Not all publications specify the absence of body parts. In the presence of an intact skull however, recognition rates for scalping on proper inspection can be expected to be high: Willey and Emerson have recorded bone defects on frontal bones from different locations at the Crow Creek massacre site between 89.2 and 94.4% [23], which may serve as minimum detection rates, assuming that all bodies had been scalped.

The overall case count for scalping in literature was low, and German literature on the topic of cDM was dominant. However, it strikes as curious that the most “typical” scalp-takings in literature, with removal of the scalp by means of a circular cut and without extensive mutilation of the face, seem to originate not from the USA, but from twentieth-century Germany, raising the question: Is scalping for the sake of criminal scalp-taking a “German phenomenon”? A plausible, albeit by no means exclusive explanation may be found in the German cultural obsession with “Indians” and the “Wild West” which persists until today [51]. It is rooted particularly in the literary works and later film adaptations of German adventure writer Karl May. In his opus magnum, the “Winnetou” series, May portrayed a noble Apache chief and his German immigrant friend “Old Shatterhand” in all sorts of adventures. Willy Wenzel’s childhood and adolescence fell into the years just after the successful release of Winnetou and in letters found at Wenzel’s, the reference is obvious with the use of terms such as “red brothers” and “pale faces”^5^ .

## Conclusion

Cases of criminal dismemberment and mutilation are rare occurrences amongst homicides, and only a fraction of those include scalping and/or scalp-taking. To our knowledge, no recent, non-historic case such as ours has so far been photo documented and published.

The overall pattern of dismemberment in our case was “defensive” and directed at enabling the transport and disposal of the victim. Apart from fine superficial cuts on the forehead and temple, no further actions were taken to destroy the victim’s identifying features. Within this pattern, the scalping was exceptional. Even though it remained unfinished, it displayed the classical characteristics of “scalping for the sake of scalp-taking” as described for and associated culturally with “North American scalp-taking”, including the presence of a “Mohawk” hairstyle. This “Mohawk” hairstyle may initially have served as a point of grabbing the obese and short-necked body by the head. The exact reasons for the progress to the mutilating act of scalping remain however speculative. Notable haemorrhage of the scalping wound indicates a perimortem or early postmortem infliction, either as a final act of maltreatment or as the first act of mutilation and dismemberment. Either way, the “Mohawk” hairstyle may have served its historical purpose of “taunting the enemy”.

## Key points

1. Scalping for the sake of scalp-taking has been performed historically in many cultures.
2. The “Mohawk” haircut is closely associated with scalping, even though not unique to North America.
3. Scalping is rare in the modern forensic context.
4. A case is presented with the attempted scalping of a homicide victim in the context of criminal dismemberment.
5. Scalping was likely attempted using a foldable hand saw, while using the “Mohawk” for grabbing.

## Data Availability

There is no data that needs to be made available.

## Footnotes

1 Non-medical disambiguations of the term “scalping” include the resale of tickets for profit, micro margin day trading, loss of flavours in packaging (food chemistry) and surface treatment of tarmac.

2 Johnston, according to oral history, went on a personal vendetta in which he killed and scalped hundreds of Crow Indians (Absaroka) in Montana.

3 William Frederick Cody, a.k.a. “Buffalo Bill”, allegedly scalped a young Cheyenne warrior during the battle of Warbonnet Creek.

4 It may be speculated that the increase of cDM cases in Sweden, observed over time, as reported in [37], reflects an increasing rate of finding bodies rather than hiding them, facilitated by factors such as cell phone coverage and rise in outdoor activities. Notably, while the total number of “offensive” mutilations remained stagnant in the publication’s cases, only the number of “defensive” mutilations (which were found) increased.

5 “Bleichgesichter”/“Blaßgesichter” refers to white people in Karl May adventure stories.
